# Porous Curdlan–Whey Protein Isolate Scaffolds Obtained by Combined Method for Cartilage Tissue Engineering Application

**DOI:** 10.3390/ma19020404

**Published:** 2026-01-20

**Authors:** Aleksandra Hnydka, Julia Higuchi, Agnieszka Grzelak, Katarzyna Klimek

**Affiliations:** 1 Chair and Department of Biochemistry and Biotechnology, Medical University of Lublin, Chodzki 1 Street, 20-093 Lublin, Poland; d.aleksandrahnydka@umlub.edu.pl (A.H.); aga.grzelakk1@gmail.com (A.G.); 2 Doctoral School, Medical University of Lublin, Chodzki 7 Street, 20-093 Lublin, Poland; 3 Laboratory of Nanostructures, Institute of High Pressure Physics, Polish Academy of Sciences, Prymasa Tysiaclecia Avenue 98, 01-142 Warsaw, Poland; j.higuchi@labnano.pl

**Keywords:** curdlan, WPI, porous scaffold, cartilage defects

## Abstract

**Highlights:**

**What are the main findings?**

- Curdlan/whey protein isolate scaffolds were fabricated using a new combined method.
- Structural, physicochemical and in vitro biological properties of scaffolds were evaluated.

**What are the implications of the main findings?**

- Fabrication method including dialysis, porogen leaching, freezing, and freeze-drying yielded porous scaffolds.
- Scaffolds showed a rough, hydrophilic structure with high absorption ability.
- Scaffold with higher protein content enhanced cell viability and proliferation in vitro.

**Abstract:**

The aim of this study was to develop porous curdlan (Cur)–whey protein isolate (WPI) biomaterials and evaluate their properties as potential cartilage scaffolds. A novel combined fabrication method involving ion-exchange dialysis, porogen leaching, freezing, and freeze-drying was employed to obtain a porous structure. Two types of scaffolds differing in protein content (5 wt.% and 7.5 wt.%) were fabricated and designated as Cur_WPI_5% and Cur_WPI_7.5%, respectively. The microstructure of the biomaterials was analyzed using stereomicroscopy and scanning electron microscopy coupled with energy-dispersive X-ray spectroscopy (SEM-EDS). Physicochemical properties, including wettability and absorption capacity, were also evaluated. In addition, the viability and proliferation of osteoblasts (hFOB 1.19 cell line) in direct contact with scaffolds were assessed. The results demonstrated that both biomaterials exhibited a porous, rough, and hydrophilic structure, as well as a high liquid absorption capacity. Cell culture studies revealed that the Cur_WPI_7.5% scaffold showed greater cytocompatibility, promoting not only osteoblast viability and but also proliferation in vitro. Overall, these findings demonstrate that the developed curdlan/WPI scaffolds, particularly Cur_WPI_7.5%, possess structural and physicochemical properties favorable for cartilage tissue regeneration, highlighting their potential as promising scaffold for future applications.

## 1. Introduction

Diseases of articular cartilage constitute a serious therapeutic challenge in modern medicine due to the limited regenerative capacity of this tissue and the increasing prevalence of degenerative joint disorders. Among them, osteoarthritis (OA) is one of the most common cartilage-related disease and a major cause of disability worldwide. The progressive degeneration of cartilage tissue leads to impaired function of the osteochondral tissue, resulting in chronic pain, joint stiffness, and reduced mobility, which significantly deteriorate patients’ quality of life [1].

Osteoarthritis can be classified into two main types: primary OA, the pathogenesis of which is not fully understood, and secondary OA, which develops as a result of identifiable factor. Predisposing conditions include previous joint injuries, congenital or developmental abnormalities, excessive physical activity, obesity, and prolonged immobilization and, for the development of osteoarthritis, include past injuries, structural abnormalities such as congenital or developmental defects, excessive physical activity, obesity, prolonged immobilization, and genetic predisposition [2]. Age is also a critical factor, as approximately 73% of individuals over 55 years are affected by OA. Moreover, epidemiological studies indicate a higher incidence of OA in women compared to men [3].

The diagnosis of OA is based on clinical evaluation and radiographic imaging, which enables the identification of characteristic features such as osteophyte formation. Disease severity is commonly assessed using the Kellgren-Lawrence grading scale, which guides the selection of appropriate treatment strategies [4]. Despite significant advances in medical science, OA remains an incurable disease, and current therapeutic approaches are primarily focuses on alleviating symptoms and slowing disease progression.

Conventional OA management includes non-pharmacological, pharmacological, and surgical interventions. Preventive and conservative treatment mainly involves physiotherapy and lifestyle modification. Pharmacotherapy relies predominantly on analgesic and anti-inflammatory agents, such as paracetamol, non-steroidal anti-inflammatory drugs, and glucocorticosteroids [5]. When conservative treatment fails to provide sufficient relief, surgical procedures are considered. Common surgical techniques include arthroscopic debridement, abrasion arthroplasty, microfracture, autologous chondrocyte implantation (ACI), and matrix-assisted chondrocyte implantation (MACI). Arthroscopic debridement is a minimally invasive procedure aimed at removing osteophytes and loose debris from the joint cavity, providing temporary pain relief but limited long-term benefits [6]. Abrasion arthroplasty and microfracture techniques stimulate bone marrow by creating perforations in the subchondral bone, inducing the release of growth factors and stem cells that can differentiate into chondrocytes [7,8,9,10,11]. However, the regenerated tissue often resembles fibrocartilage rather than native hyaline cartilage, which compromises its durability [12].

Advanced regenerative approaches, such as ACI and MACI, aim to restore cartilage defects more effectively. ACI is a two-stage procedure involving the isolation, in vitro expansion, and subsequent implantation of autologous chondrocytes into defect site [13]. MACI represents a further development of this technique, in which chondrocytes are seeded onto a biomaterial prior to implantation, improving cell distribution and surgical handling while enhancing patient tolerance [14,15].

In this context, tissue engineering has emerged as a promising strategy for cartilage regeneration. Biomaterials designed for cartilage repair aim to mimic the native extracellular matrix, thereby supporting cell adhesion, proliferation, and differentiation in a three-dimensional environment. An ideal scaffold should be biocompatible, possess appropriate mechanical properties, exhibit controlled biodegradation into non-toxic by-products, and contain a porous structure that facilitates nutrient diffusion and tissue ingrowth [16,17]. Furthermore, modern biomaterials are increasingly expected to actively regulate cellular behavior and promote tissue-specific regeneration [18]. The composition, structure, and fabrication method of biomaterials play a crucial role in determining their final properties and biological performance [19]. Consequently, there is a growing interest in the development of novel scaffolds or the modification of existing materials to improve their suitability for cartilage tissue engineering.

In this study, novel curdlan/WPI biomaterials are fabricated using an innovative combined processing technique. Their structural, physicochemical, and biological properties were systematically investigated to assess their potential application as scaffolds for cartilage tissue regeneration.

## 2. Materials and Methods

### 2.1. Materials

Bovine serum albumin (BSA), calcium carbonate (CaCO_3_), calcium chloride (CaCl_2_), Cell Counting Kit (WST-8), DMEM/Ham’s F12 medium, G418 disulfate salt, Hoechst 33342 fluorescent dye, HPLC grade water, L-glutamine solution, a Live/Dead Cell Double Staining Kit, sodium chloride (NaCl), penicillin-streptomycin solution, phosphate-buffered saline (PBS), trypsin-EDTA solution 0.25%, triton X-100, were obtained from Sigma-Aldrich Chemicals (now Merck S.A.), Warsaw, Poland. Curdlan was purchased from Wako pure Chemicals Industries, Osaka, Japan, whereas the fetal bovine serum (FBS) was from Pan-Biotech, Aidenbach, Germany. Normal human fetal osteoblasts (hFOB 1.19, ATCC CRL-3602) were supplied from American Type Culture Collection (ATCC), Manassas, VA, USA. AlexaFluor635-phalloidin fluorescent dye was purchased from Invitrogen, Warsaw, Poland, while hydrochloric acid (HCl) and sodium hydroxide (NaOH) were from POCH, Gliwice, Poland. Whey protein isolate (WPI) was obtained from Agropur Cooperative, Eden Prairie, MN, USA.

### 2.2. Methods

#### 2.2.1. Fabrication of Curdlan/WPI Biomaterials

The fabrication procedure of the developed biomaterials is described in patent application no. P.453835 entitled “Porous biomaterial based on curdlan and whey protein isolate for application in the treatment of cartilage and osteochondral defects and its preparation method”. The procedure began with the formulation of a curdlan solution. Curdlan was dissolved at a concentration of 0.12 g/mL in a 0.3 M sodium hydroxide (NaOH) solution. In parallel, the WPI solution was prepared. Briefly, 0.10 g and 0.15 g of WPI were weighed into separate 2 mL Eppendorf tubes and each sample was suspended in 1 mL of distilled water. The protein suspensions were incubated at 93 °C for 15 min to induce protein denaturation. After incubation, the solutions were cooled to room temperature and subsequently mixed with the alkaline curdlan solution to obtain final formulations containing 6 wt.% of curdlan with either 5 wt.% or 7.5 wt.% of WPI.

To generate porosity, calcium carbonate (CaCO_3_) was used as a porogenic agent. An amount of 0.5 g of CaCO_3_ was added to each formulation and thoroughly mixed to ensure homogeneous distribution. The resulting mixtures were transferred into dialysis tubing and were immersed in a 10% calcium chloride (CaCl_2_) solution, and dialyzed for 24 h at room temperature. Following dialysis, the formed biomaterials were rinsed with distilled water, cut into small pieces (approximately 2–3 mm), and transferred to Falcon tubes containing 0.5 M hydrochloric acid (HCl) to remove the porogen. After complete elution of CaCO_3_, the samples were thoroughly washed with distilled water to remove residual acid. The biomaterials were then placed in Petri dishes, frozen at −20 °C for 24 h, and subsequently freeze-dried. The scaffolds were packaged in plastic–paper sterilization pouches and sterilized with ethylene oxide at 55 °C for 5 h.

**Figure 1:**
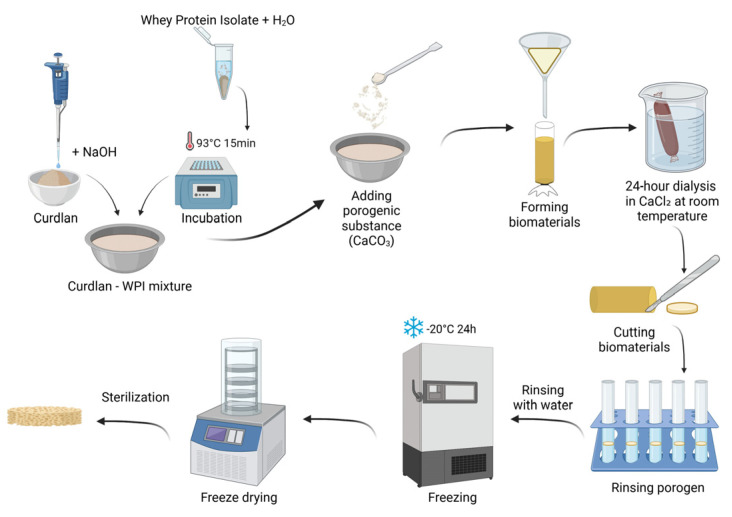
Fabrication process of biomaterials according to patent application no. P.453835. Created in Biorender. Klimek K. (2026) https://BioRender.com/mo3m2g9 (accessed on 15 January 2026).

The biomaterial containing 5 wt.% of WPI was designated as Cur_WPI_5%, while the biomaterial with 7.5 wt.% of WPI was denoted as Cur_WPI_7.5%. The sterilized scaffolds were used for further structural, physicochemical, and biological characterization. Figure 1 presents fabrication process of biomaterials.

#### 2.2.2. Evaluation of the Structural Properties of Biomaterials

A stereoscopic microscope (Olympus SZ61TR, Olympus, Warsaw, Poland) and SEM (Ultra Plus GEMINI, Carl Zeiss, Jena, Germany) equipped with energy-dispersive X-ray spectroscopy (EDX) system (EDX Quantax 400, Bruker, MS, USA) were used to examine the morphology of the obtained biomaterials. Before analysis, the samples were coated with a nanometric layer of a gold/palladium (80/20) or carbon mixture. An accelerating voltage of 2–3 kV was used for observation.

Topographic analysis of the biomaterials was performed using a digital microscope (VHX-7000, Keyence, Osaka, Japan) with a DIC (VH-Z100UT) varifocal lens for roughness profile measurement. A magnification of 200× was used. The images were exported as three-dimensional reconstructions, on which profilometric analysis was subsequently performed.

#### 2.2.3. Evaluation of Physicochemical Properties of Biomaterials

To assess the wettability of biomaterial’s surface, the water contact angle (WCA) was measured in contact with HPLC-grade water (DSA25B, Krüss, Hamburg, Germany). Measurements were taken in two environments: in open air and under controlled conditions, where the temperature was 22 °C and the humidity was 40%. The WCA was measured using a syringe equipped with a needle and the analysis consisted of placing one drop with a volume of 10 µL on the sample at 10 s intervals. During the test, digital measurements were taken using Krűss ADVANCE software based on the geometric method.

The liquid absorption test began with the weighing of dry samples of the biomaterials. Four samples of biomaterials (*n* = 4) were used for analysis. After that, a 0.9% sodium chloride (NaCl) solution was poured into the 12-wells plate. The tested samples were placed in wells containing the sodium chloride solution and then were weighed after 3, 6, 9, 12, 15, 25, 35, 45, 60, 120, and 1440 min. After the appropriate amount of time had elapsed, the mass was measured by removing the biomaterial from the 0.9% NaCl solution with tweezers, draining the excess solution, and weighing it on a weighing dish. After weighing, the biomaterials were re-immersed in the solution in the well of the plate.

The ability to absorb fluids was calculated using the following equation:

$$PW=\frac{Wt-Ws}{Ws}*100$$

where

PW—increase in biomaterial mass; Wt—mass of wet biomaterial at time t; Ws—mass of dry biomaterial at time 0.

#### 2.2.4. Evaluation of Cytocompatibility of Biomaterials

The hFOB 1.19 cell line (cat. no. CRL-3602) from the ATCC was used for the experiments as a model cells. The hFOB 1.19 cells were grown in DMEM/Ham F-12 medium supplemented with 10% fetal bovine serum, 300 µg/mL of G418 antibiotic, 100 U/mL of penicillin, and 100 µg/mL of streptomycin. The culture medium did not contain phenol red. The culture temperature was 34 °C and the CO_2_ concentration was 5%.

##### Cell Viability Assessment

Cells were seeded directly onto biomaterials at a density of 2 × 10^5^ cells/mL and then incubated at 34 °C, 95% humidity, and 5% CO_2_. Cell viability was assessed after 24 and 48 h of incubation using a Live/Dead Doubling Staining Kit and determined based on confocal laser scanning microscope (CLSM, Fluoview FV-1000 confocal microscope (Olympus, Shinjuku, Japan) observations—live cells emitted green fluorescence, while the dead cells were visible as red fluorescence. Imaris 7.2.3 software (Bitplane, Olympus, Shinjuku, Japan) was used to reconstruct the microscopic images.

##### Assessment of Cell Proliferation Using the WST-8 Assay

Cells were seeded directly onto biomaterials at a density of 1 × 10^5^ cells/mL and were incubated at 34 °C, 95% humidity, and 5% CO_2_. The culture medium was replaced with fresh medium every 3 days. Cell proliferation was quantitatively assessed after 4 and 7 days using the WST-8 assay, according to the manufacturer’s protocol. Optical density (OD) was measured at 460 nm, with background subtraction at 650 nm. OD values are directly proportional to the number of metabolically active cells, allowing monitoring of proliferation over time. Four independent samples of biomaterials (*n* = 4) were used in the experiment.

##### Morphology Assessment Using Actin Cytoskeleton and Nuclei Staining

After the WST-8 test, the same samples were fixed with a 3.7% formaldehyde solution for 10 min at room temperature, permeabilized with 0.2% Triton X-100 solution in PBS for 5 min, and blocked with 1% BSA solution in PBS for 20 min at room temperature. Cells were then stained with 1 µg/mL Hoechst 33342 for nuclei and 2 U/mL phalloidin conjugated with Alexa Fluor 635 fluorochrome for the actin cytoskeleton. Cell nuclei emitted blue fluorescence, while the cell cytoskeleton was visible as red fluorescence. The stained cells were observed using a Fluoview FV-1000 CLSM (Olympus, Shinjuku, Japan). The images were reconstructed using Imaris 7.2.3 software (Bitplane, Shinjuku, Japan).

#### 2.2.5. Statistical Analysis

All experiments were performed in at least three independent replicates, and the data were presented as mean value ± standard deviation (SD). Data normality was assessed using the D’Agostino–Pearson omnibus test. Statistical analyses were conducted using either an unpaired Student’s *t*-test or One-way or Two-way analysis of variance (ANOVA), followed by post hoc multiple comparison test. Differences were considered statistically significant at *p* < 0.05. Statistical analyses were performed using GraphPad Prism software (version 5.04; GraphPad Software, San Diego, CA, USA).

## 3. Results

### 3.1. Structural Properties of Curdlan/WPI Biomaterials

#### 3.1.1. Microstructure Imaging

**Figure 2:**
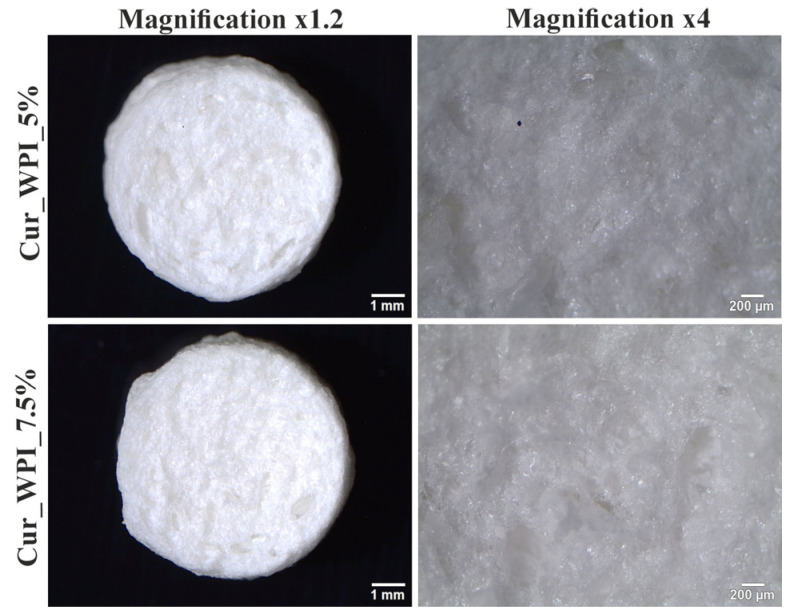
Microstructure of biomaterials Cur_WPI_5% and Cur_WPI_7.5% imaged using a stereoscopic microscope (magnification 1.2×, bar scale = 1 mm and 4×, bar scale = 200 µm).

Morphological imaging using a stereoscopic microscope showed that both biomaterials possessed a porous and rough structure, as can be seen in Figure 2.

**Figure 3:**
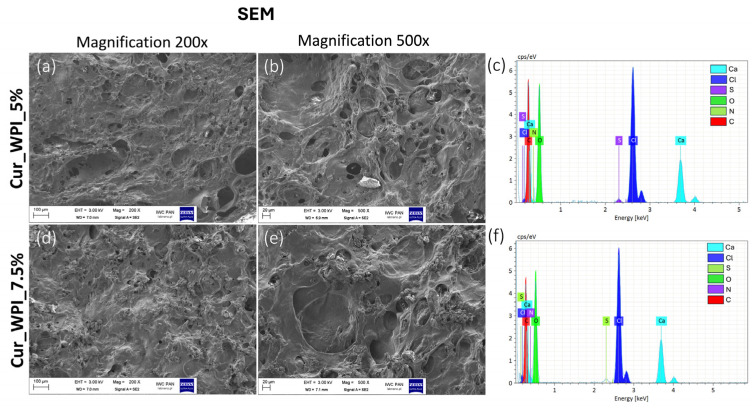
SEM-EDS analysis of biomaterials: Cur_WPI_5% (**a**–**c**) and Cur_WPI_7.5% (**d**–**f**). Magnification 200×, bar scale = 100 µm; magnification 500×, bar scale = 20 µm.

Observations using SEM confirmed that both biomaterials had a porous structure with clearly visible pores (Figure 3a,b,d,e). In addition, EDS analysis was used to identify the elemental composition of the biomaterials. In addition to elements characteristic of polysaccharides and proteins, such as nitrogen (N), oxygen (O), sulfur (S), and carbon (C), the presence of calcium (Ca) and chlorine (Cl) was also noted (Figure 3c,f). Most probably, the presence of calcium and chlorine in the structure of biomaterials is the result of their production process, which involves ion-exchange dialysis with calcium chloride solution.

#### 3.1.2. Surface Roughness

**Figure 4:**
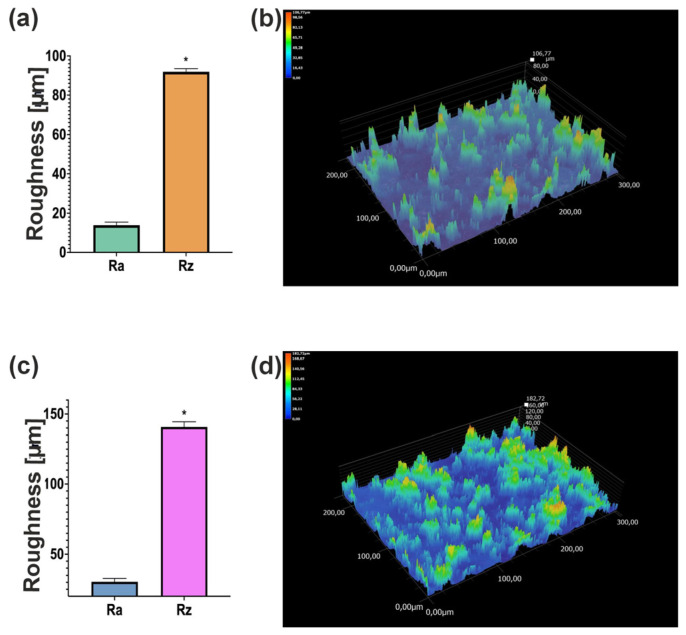
Surface roughness of Cur_WPI_5% (**a**,**b**) and Cur_WPI_7.5% (**c**,**d**) biomaterials. * Statistically significant difference between the highest roughness profile height (Rz) and the arithmetic mean of the roughness profile ordinates (Ra) based on Student’s *t*-test, *p* < 0.05.

Analysis of the biomaterial topography (Figure 4) showed that both biomaterials had a rough structure. In the case of the Cur_WPI_5% biomaterial, the arithmetic mean of the roughness profile ordinates (Ra) was 14 μm, and the highest roughness profile height (Rz) was approximately 92 μm. In contrast, for the Cur_WPI_15% biomaterial, these values were 30 μm and 141 μm, respectively. The difference between the Ra and Rz values was statistically significant in both groups.

### 3.2. Physicochemical Properties of Biomaterials

#### 3.2.1. Surface Wettability

**Figure 5:**
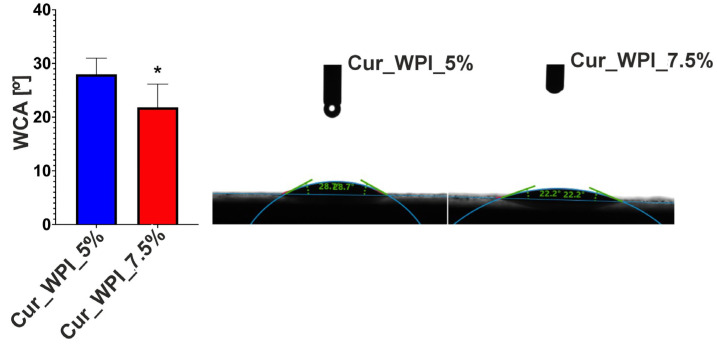
Surface wettability expressed as the WCA for Cur_WPI_5% and Cur_WPI_7.5% biomaterials. * Statistically significant difference compared to biomaterial Cur_WPI_5% based on Student’s *t*-test, *p* < 0.05.

The test showed that the surfaces of both biomaterials had a WCA less than 30° (Figure 5). The WCA of the Cur_WPI_7.5% biomaterial was statistically significantly lower (approximately 22°) compared to the WCA of the Cur_WPI_5% biomaterial (approximately 28°).

#### 3.2.2. Liquid Absorption Capacity

**Figure 6:**
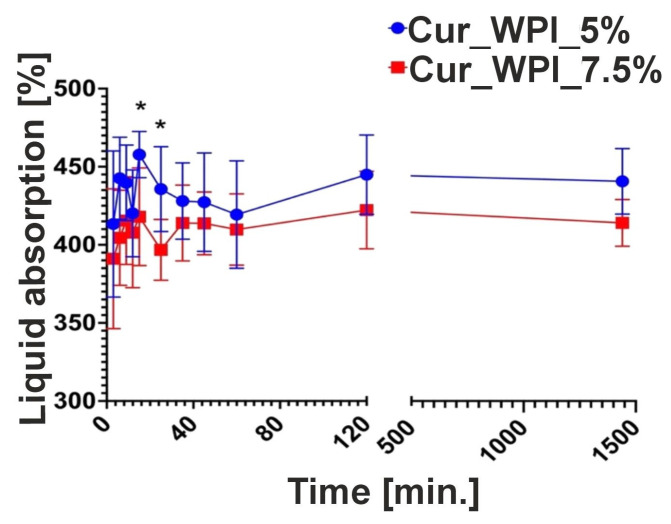
The ability of Cur_WPI_5% and Cur_WPI_7.5% biomaterials to absorb liquids expressed as percentage weight gain—PW [%] after soaking in a 0.9% NaCl solution. * Statistically significant difference compared to the Cur_WPI_5% biomaterial based on a one-way ANOVA test, followed by Tukey’s multiple comparison test, *p* < 0.05.

According to measurements of the weight of biomaterials (Figure 6) after incubation with a 0.9% NaCl solution for 1440 min, both materials were found to have a high liquid absorption capacity. In addition, the Cur_WPI_5% biomaterial was able to absorb a greater amount of solution than the Cur_WPI_7.5% biomaterial. The largest percentage increase in the mass of the Cur_WPI_5% biomaterial was observed after 15 min, while for the Cur_WPI_7.5% biomaterial, it was observed after 120 min.

### 3.3. Cytocompatibility of Biomaterials

**Figure 7:**
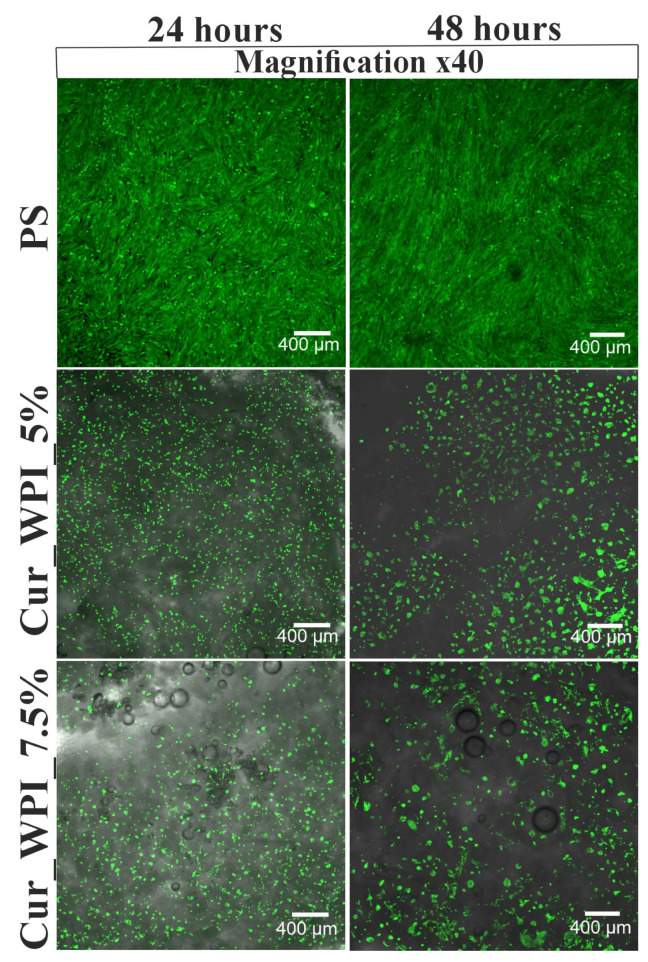
Confocal microscope images showing the growth of hFOB 1.19 cells colonizing the surfaces of Cur_WPI_5% and Cur_WPI_7.5% biomaterials and polystyrene (PS) after 24 and 48 h incubation. Green fluorescence was generated by living cells, red fluorescence was emitted by dead cells. Magnification 40×, bar scale = 400 µm.

Cell viability assessment showed that PS (polystyrene—control), Cur_WPI_5%, and Cur_WPI_7.5% biomaterials supported cell growth (Figure 7). After both 24 and 48 h of incubation, most cells emitted green fluorescence, indicating their high viability. No red fluorescence corresponding to dead cells were observed under the applied imaging conditions, confirming the no cytotoxicity of the biomaterials. Both green and red channels were used during image acquisition, and the absence of red signal reflects the actual cell viability rather than imaging setting.

**Figure 8:**
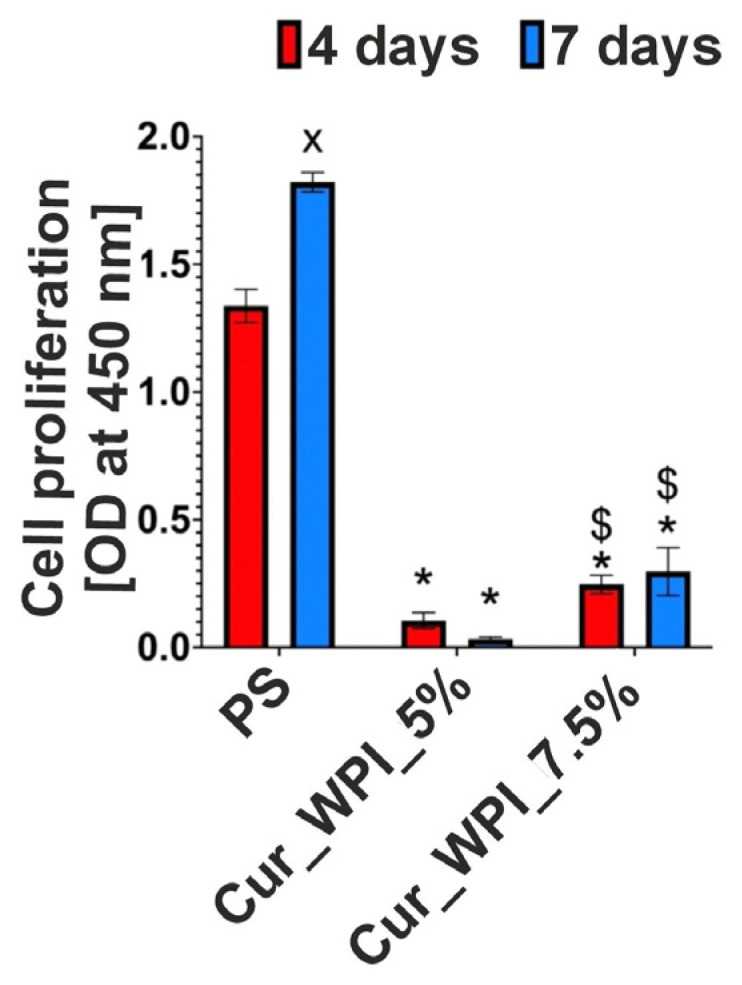
Evaluation of hFOB 1.19 cell proliferation growing on Cur_WPI_5% and Cur_WPI_7.5% biomaterials and polystyrene (PS—control). OD values were determined in the WST-8 test after 4 and 7 days of incubation. * Statistically significant difference compared to the control after both 4 and 7 days of incubation; ^x^ Statistically significant difference compared to day four; ^$^ Statistically significant difference between the Cur_WPI_7.5% biomaterial and the Cur_WPI_5% biomaterial; two-way ANOVA test, followed by a Bonferroni comparison test; *p* < 0.05.

The WST-8 test showed that the amount of hFOB 1.19 cells growing on both polystyrene (PS—control) and Cur_WPI_7.5% biomaterial increased over time (Figure 8). After 4 and 7 days of incubation, the OD values were 1.32 and 2.19 for PS and 0.25 and 0.32 for the Cur_WPI_7.5% biomaterial, respectively. Therefore, their surfaces promoted cell division over time. In the case of the Cur_WPI_5% biomaterial, it was noted that the OD values decreased with increasing incubation time (OD = 0.1 after 4 days and OD = 0.033 after 7 days), which indicates that the surface of this biomaterial does not promote hFOB 1.19 cell division over time.

**Figure 9:**
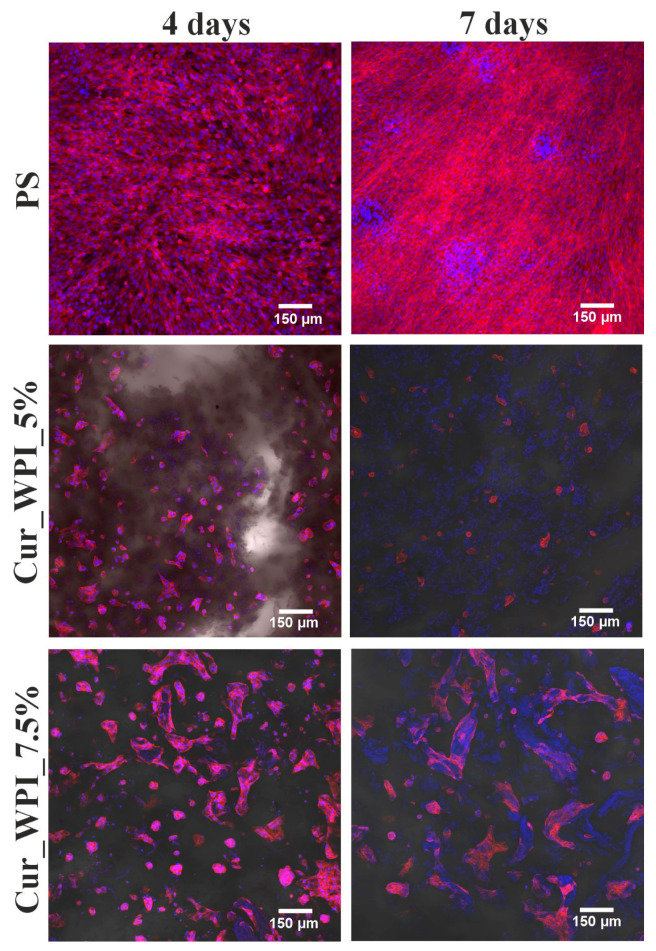
Confocal microscope images showing the morphology of hFOB 1.19 cells growing on the surfaces of Cur_WPI_5% and Cur_WPI_7.5% biomaterials and polystyrene (PS—control) for 4 and 7 days. Cell nuclei are visible as blue fluorescence, while actin filaments of the cytoskeleton are visible as red fluorescence. Magnification 100×, scale bar = 150 µm.

Qualitative analysis using a confocal microscope (Figure 9) provided additional insight into cell behavior on the investigated surfaces. While nuclei were visible on all samples, these observations should not be interpreted as direct evidence of cell proliferation. Cells cultured on PS and Cur_WPI_7.5% exhibited a well-spread morphology with clearly organized actin cytoskeleton, indicating good cell attachment and spreading. In contrast, cells on Cur_WPI_5% appeared less flattened, with weaker and less organized actin filament staining and more prominent nuclei, which is characteristic of poorly attached or weakly spread cells.

Therefore, the observations in Figure 9 primarily reflect differences in cell morphology, adhesion, and cytoskeletal organization in response to the scaffold surfaces.

## 4. Discussion

Treatment options for osteoarthritis are limited by the low self-regeneration ability of cartilage tissue. Therefore, one promising option is tissue engineering. Scientists are exploring the possibility of replacing the cartilage defect with a three-dimensional biomaterial that facilitates cell proliferation and differentiation [20]. Currently, various biomaterials are used, which are available on the medical market. However, due to the increasing number of patients affected by osteoarthritis, new solutions are being sought to support the reconstruction of damaged cartilage [21].

Therefore, two new porous biomaterials (Cur_WPI_5% and Cur_WPI_7.5%) were developed based on curdlan and WPI. Both biomaterials contained 6 wt.% of curdlan but differed in the protein content. Cur_WPI_5% contained 5 wt.% of WPI, and Cur_WPI_7.5% contained 7.5 wt.% of WPI. The resulting scaffolds were analyzed for their structural, physicochemical, and biological properties.

Microstructure imaging revealed that both biomaterials contained pores (Figure 2). It was assumed that a combined method consisting of ion-exchange dialysis, porogen washout, freezing, and freeze-drying would achieve this structure. Using calcium carbonate as the porogen and then washing it out allowed for the formation of free spaces (pores) [22]. Moreover, freezing and freeze-drying also contributed to the obtained porosity, by dehydrating the biomaterials—converting the solvent into ice and then sublimating [23]. Consequently, the use of this production method resulted in the formation of visible pores on the surface of the biomaterials (Figure 3a,b,d,e). Meanwhile, EDS analysis revealed the presence of chlorine and calcium (Figure 3c,f). The result of this test was consistent with the purpose, as ion exchange dialysis was used in the production of biomaterials. According to current knowledge, calcium ions promote cell adhesion and proliferation [24].

The observation of biomaterials using a digital microscope showed that scaffolds have a rough surface (Figure 4). This is related to the presence of pores in their structure. However, the surface of the Cur_WPI_7.5% biomaterial was more porous than that of Cur_WPI_5%. This is interesting because both scaffolds had the same amount of curdlan and porogenic substance. It turns out that the amount of protein component affects the roughness of their surface. Abraham et al. observed a similar effect in their study, where the roughness of biomaterials increased with the amount of collagen [25]. It is important to highlight that surface area is an important aspect in the design of biomaterials. Appropriate roughness influences cellular activity by promoting cell adhesion and the response of surrounding tissue to the implanted biomaterial [26]. Therefore, Cur_WPI_7.5% should show better biological properties.

Surface wettability analysis, which involves examining the angle between the material and liquid, e.g., water (water contact angle—WCA), allows for the determination of the biomaterial’s behavior with surrounding fluids. The surface can be hydrophilic or hydrophobic [27]. It is considered that hydrophilicity, i.e., high wettability (contact angle 0–90°), indirectly supports cell migration, adhesion and proliferation [28]. This is related to the increased ability to adsorb proteins contained in culture fluids (in vitro) or body fluids (in vivo) to which cells migrate. The analysis showed that both Cur_WPI_5% and Cur_WPI_7.5% have a hydrophilic surface (WCA below 30°) (Figure 5). This feature should potentially promote better cell colonization. It is worth noting that biomaterial with a higher protein content exhibits greater surface wettability. This is because proteins are highly hydrophilic molecules. This relationship was also demonstrated by Ahmed et al. in their study, which was based on the production of cartilage biomaterials based on polylactide and polylactide-glycolide copolymer with the addition of collagen. It was shown that an increase in the protein content of the composites contributed to a reduction in their contact angle (i.e., increasing wettability) [29].

The ability of biomaterials to absorb fluids was also assessed. This test involves measuring the time required for the biomaterial to become saturated. This is an important aspect of developing new biomaterials, as implantable biomaterials need to soak in a suitable solution (e.g., saline or the patient’s blood) before application. It is important to note that excessive saturation time may induce inflammation and increase fluid diffusion from surrounding tissues [30]. It is thought that biomaterials can be excluded within 30 min [31]. Cur_WPI_5% and Cur_WPI_7.5% had the highest fluid absorption capacity for up to 30 min (Figure 6). This high fluid absorption capacity is related to their hydrophilic properties.

The cytocompatible properties of the developed biomaterials were examined by viability (Figure 7) and proliferation (Figure 8 and Figure 9) assessments. In both tests, polystyrene (PS—control) supported cell viability/division to a greater extent than the developed biomaterials. However, it is widely known that this is due to the fact that cells need much less time to adapt to the flat surface of polystyrene than to the three-dimensional surface of biomaterials. [32]. Despite the fact that the Cur_WPI_5% biomaterial exhibited beneficial structural properties and a hydrophilic surface that promote cell viability, it was found that it did not support cell proliferation. In contrast, the Cur_WPI_7.5% biomaterial, with a similar structure and physicochemical properties, supported both cell viability and proliferation over time. This means that the difference in protein content in the biomaterials significantly affected the cellular response. Due to the fact that Cur_WPI_7.5% exhibits higher cytocompatibility compared to Cur_WPI_5%, it can be considered a biomaterial that can potentially be used for the regeneration of cartilage defects resulting from osteoarthritis.

## 5. Limitations of the Study and Future Perspectives

Despite the promising results obtained in this study, several limitations should be acknowledged. First, the porosity of the scaffolds was evaluated only qualitatively using SEM. Quantitative analysis of pore size, distribution, and interconnectivity is necessary in future studies to better understand the relationship between scaffold structure and cellular responses.

Second, cytocompatibility was assessed using osteoblasts (hFOB 1.19), which, although useful for general evaluation of cell–material interactions, are not the native cells of cartilage tissue. Primary human chondrocytes, ideally isolated from orthopedic patients, should be employed in future in vitro studies to more accurately assess the potential of these biomaterials for cartilage regeneration.

Finally, all experiments were performed in vitro, which cannot fully replicate the complex physiological environment of cartilage, including mechanical loading, biochemical signaling, and tissue interactions. Therefore, in vivo studies are essential to confirm biocompatibility, integration, and functional performance of the scaffolds as implantable biomaterials.

Overall, while this study provides an initial evaluation of the structural, physicochemical, and biological properties of Cur/WPI biomaterials, further studies are required to fully demonstrate their potential as scaffolds for cartilage tissue engineering.

## 6. Conclusions

Considering the request for new therapeutic options for osteoarthritis, there is a growing interest in tissue engineering. Biomaterials are being designed that provide three-dimensional scaffolds for cells and reflect the natural extracellular matrix, allowing for increased cartilage tissue regeneration. In this study, two curdlan–whey protein isolate biomaterials (Cur_WPI_5% and Cur_WPI_75%) were presented, which differed in their protein content. The biomaterials were fabricated using an innovative, combined method involving ion-exchange dialysis, porogen leaching, freezing, and freeze-drying. Both scaffolds were characterized by the presence of pores in their structure, a rough surface, and a hydrophilic nature. It was shown that the increase in protein content in the composite contributed to the increased cytocompatibility of the Cur_WPI_7.5% biomaterial, potentially making it a more promising implantable biomaterial. To confirm the validity of using Cur_WPI_7.5% in the treatment of cartilage defects, further tests are necessary, with reference to in vivo tests and then clinical trials.

## 7. Patents

The fabrication procedure of the developed biomaterials is described in patent application no. P.453835 entitled “Porous biomaterial based on curdlan and whey protein isolate for application in the treatment of cartilage and osteochondral defects and its preparation method”.

## Data Availability

Data are available on reasonable request. The data can be obtained from A.H., J.H. and K.K.

## Abbreviations

The following abbreviations are used in this manuscript:

ACI	Autologous chondrocyte implantation
BSA	Bovine serum albumin
CaCl_2_	Calcium chloride
CaCO_3_	Calcium carbonate
CLSM	Confocal laser scanning microscope
CO_2_	Carbon dioxide
Cur	Curdlan
EDS	Energy-dispersive X-ray spectroscopy
FBS	Fetal bovine serum
HCl	Hydrochloric acid
HPLC	High-performance liquid chromatography
MACI	Matrix-assisted chondrocyte implantation
NaCl	Sodium chloride
NaOH	Sodium hydroxide
OA	Osteoarthritis
OD	Optical density
PBS	Phosphate-buffered saline
PS	Polystyrene
PW	Increase in biomaterial mass
Ra	Arithmetic mean of the roughness profile ordinates
Rz	Highest roughness profile height
SD	Standard deviation
SEM	Scanning electron microscopy
T	Time
WCA	Water contact angle
WPI	Whey protein isolate
Ws	Mass of dry biomaterial at time 0
Wt	Mass of wet biomaterial at time t
